# Cognitive function during early abstinence from opioid dependence: a comparison to age, gender, and verbal intelligence matched controls

**DOI:** 10.1186/1471-244X-6-9

**Published:** 2006-02-24

**Authors:** Pekka Rapeli, Reetta Kivisaari, Taina Autti, Seppo Kähkönen, Varpu Puuskari, Olga Jokela, Hely Kalska

**Affiliations:** 1 Psychiatric unit for drug dependence, Department of Psychiatry, Helsinki University Central Hospital, Finland; 2 Unit on Prevention and Treatment of Addictions, Department of Mental Health and Alcohol Research, National Public Health Institute, Finland; 3 Medical Imaging Center, Helsinki University Central Hospital, Finland; 4 BioMag Laboratory, Engineering Center, Helsinki University Central Hospital, Finland; 5 Cognitive Brain Research Unit, University of Helsinki, Finland; 6 Department of Psychiatry, Helsinki University Central Hospital, Finland; 7 Department of Psychology, University of Helsinki, Finland

## Abstract

**Background:**

Individuals with opioid dependence have cognitive deficits during abuse period in attention, working memory, episodic memory, and executive function. After protracted abstinence consistent cognitive deficit has been found only in executive function. However, few studies have explored cognitive function during first weeks of abstinence. The purpose of this study was to study cognitive function of individuals with opioid dependence during early abstinence. It was hypothesized that cognitive deficits are pronounced immediately after peak withdrawal symptoms have passed and then partially recover.

**Methods:**

Fifteen patients with opioid dependence and fifteen controls matched for, age, gender, and verbal intelligence were tested with a cognitive test battery When patients performed worse than controls correlations between cognitive performance and days of withdrawal, duration of opioid abuse, duration of any substance abuse, or opioid withdrawal symptom inventory score (Short Opiate Withdrawal Scale) were analyzed.

**Results:**

Early abstinent opioid dependent patients performed statistically significantly worse than controls in tests measuring complex working memory, executive function, and fluid intelligence. Their complex working memory and fluid intelligence performances correlated statistically significantly with days of withdrawal.

**Conclusion:**

The results indicate a rather general neurocognitive deficit in higher order cognition. It is suggested that cognitive deficit during early abstinence from opioid dependence is related to withdrawal induced neural dysregulation in the prefrontal cortex and is partly transient.

## Background

When opioids are used chronically as pain relievers they may cause mild cognitive deficits in attention, complex working memory, and episodic memory [1,2]. So far, there is no evidence that these effects would continue after cessation of oral opioid medication [3]. However, when opioids are abused, they are often used intravenously with frequent high doses and in parallel with other drugs of abuse. Not surprisingly, cognitive associations of chronic opioid abuse are more pronounced than in medical use. In studies concerning very recent or ongoing chronic opioid abuse there is even some, though not consistent, evidence for general intellectual decline [4,5]. More consistently, there is evidence for deficits in attention, working memory, memory, and executive function [6-8]. Again, the studies concerning abstinence from opioid abuse show better cognitive function than during opioid abuse [9-14]. However, there are important differences between early and late opioid abstinence, and these need more consideration. Vast majority of relapses back to opioid abuse in out-patient treatment settings take place during first weeks of abstinence [15,16]. Thus, the study of cognitive function within this time is relevant.

During early opioid abstinence somatic signs of withdrawal are first easily noticed and then gradually disappear. These include flu-like symptoms and changes in heart rate and blood pressure. The symptoms typically peak within three days from the last dose of intravenous heroin and within five days from the last dose of intravenous buprenorphine [17,18]. Instead, during late abstinence, after first three weeks, somatic signs of opioid withdrawal are seldom observed but drug craving may still be present.

Early opioid abstinent patients often complain fatigue and poor concentrating. In agreement with this a cascade of neural dysregulations takes place during that time. The cascade starts with abrupt downregulation of mu opiate receptor activity together with elevation of gamma-aminobutyric acid (GABA) and dynorphin release in the striatum and the limbic system [19]. This is followed by increased release of noradrenaline in the locus couruleus and the bed nucleus of the stria terminalis paired with excessive glutamate release in the hippocampus and the anterior cingulate cortex. Marked reduction of dopamine activity in the mesolimbic system and its projections has an inverse relationship with a strong increase of dopamine, noradrenaline and serotonin content in the medial prefrontal cortex [20]. Finally, brain stress systems are activated due to noradrenergic activation of corticotrophin releasing factor from the hypothalamus and the amygdala [21] In concordance with this an elevation of cortisol secretion takes place during early opioid abstinence [22]. Against this background, it is surprising that cognitive function during early opioid abstinence has been investigated only in one study.

In a study by Guerra et al. individuals with current heroin abuse showed deficits in attention, working memory, episodic memory, and verbal fluency [9]. At retest 7–14 days after admission to rapid detoxification treatment their performance reached the level of controls. The results indicate a rapid recovery of cognitive function. However, practice effects were not controlled for.

There are more studies concerning cognitive function during late opioid abstinence. In Gerra et al. study patients with opioid abuse history were studied four months from detoxification [10]. Patients with antisocial personality disorder showed deficits in complex attention and in executive function. Other patients showed no deficits. In Davis et al. study patients with six months of opioid abstinence showed no specific cognitive deficits [11]. In Lee and Pau study previous heroin users with eight months of abstinence as well as in Pau et al. study with fourteen months of abstinence showed deficit in executive function [12,13] In Mintzer et al. study patients with nine months of opioid abstinence showed normal cognitive performance expect in one task combining visual attention and flexibility [14]. Thus, in studies concerning late opioid abstinence executive function deficit has been most consistent finding.

The main aim of the present study was to explore cognitive function of individuals with opioid dependence during early abstinence in comparison to normal controls. The groups were matched for age, gender, and verbal intelligence (VIQ). On the basis of opioid withdrawal related neural cascade and earlier research deficits in working memory, episodic memory, and in executive function were expected. Non-optimal catecholamine levels in prefrontal cortex may cause working memory deficit, especially when together with hypercortisolism [23,24]. Executive function may be impaired because it is dependent on optimal noradrenaline function [25,26]. Also learning and memory may be impaired. Chronic exposure to opiates reduces the birth of new neurons in the dentate gyrus of the adult hippocampus, and this is known to affect learning and memory [27,28]. In addition, fluid intelligence was measured. It is known to be more sensitive to frontal lobe dysfunction than conventional executive function tests [29-31]. Therefore, it was hypothesized that also fluid intelligence performance would be reduced.

Our second aim was to explore if cognitive deficits in early opioid abstinence are at least partially transient. Opioid-induced neuronal changes in the prefrontal cortex, hippocampus, or thalamus show signs of recovery within first month of cessation of opioid treatment [32]. Abnormal elevations of neural activity in the striatum and the amygdala reduce within 1 – 2 first weeks from opioid withdrawal [19]. In concert with this, high cortisol levels resume to normal levels within first weeks of abstinence [33]. Normal function of the catecholaminergic and GABAergic systems after few months of opioid abstinence has been reported with some exceptions concerning psychiatric co-morbidity [10,34-36]. Thus, the hypothesized cognitive deficits may show recovery. Therefore, we analyzed correlations between cognitive performance and days of withdrawal; and between cognitive performance and subjective opioid withdrawal symptoms, whenever opioid dependent individuals performed worse than controls. In addition, as long-term neurotoxicity related to substance abuse is possible, correlations between duration of opioid abuse or any substance abuse and cognitive performance were analyzed whenever patients performed worse than controls.

## Methods

The participants of the study included 15 individuals with opioid dependence who were voluntary inpatients from Helsinki University Central Hospital drug detoxification unit and 15 controls. All participants were between ages 20 – 50 years. Participants with uncontrolled mixed substance abuse, acute alcohol abuse, or acute axis I psychiatric morbidity according to Diagnostic and Statistical Manual of Mental Disorders (DSM-IV) not related to substance were excluded. Also participants with severe brain injury, chronic neurological disease, with history of epileptic seizures, with human immunodeficiency virus (HIV), primary organic cognitive deficit, or magnetic objects contraindicative for magnetic resonance imaging (MRI) were excluded from the study. Each participant was evaluated by brain MRI, and participants showing lesions indicating vascular pathology or brain injury were excluded. The study protocol was accepted by the Ethics Committee of Helsinki University Central Hospital. A written informed consent according to the Declaration of Helsinki was obtained from all participants.

All participants with opioid dependence were voluntary patients from a series of consecutive patients admitted for potential methadone maintenance treatment. The patients were hospitalized for two weeks in a drug withdrawal unit before starting methadone substitution treatment. The criteria for this in our institute were a minimum age of 20 years, four years of documented opioid dependence, and failure of institutional or long-lasting outpatient opioid withdrawal. In the patient group there were several cases for all variants of hepatic viruses (A, B, or C). However, none of these were in acute phase. All participants that were studied for HIV were negative. One patient refused to be tested for HIV. The patients had no neurological complains. Two patients either refused to be tested or stopped inpatient treatment.

A control group matched for age, gender, and VIQ was recruited from the staff of our institution. The VIQ matching was based on Wechsler's revised intelligence scale (WAIS-R) vocabulary subtest [37]. None of the controls had abused illegal drugs, but all of them had taken alcohol on social occasions. However, none of them met the criteria of abuse or dependence on alcohol. The controls were screened by psychiatric interview of having no history of major psychiatric morbidity or substance abuse. Demographic variables of the groups are presented in Table 1. As a group the controls had more education than participants with opioid dependence.

**Table 1:** Group demographics

	**Participants with opioid dependence (n = 15)**	**Controls (n = 15)**	**P-value**
Age, years (*M, SD*)	31.6 (5.8)	31.3 (5.9)	NS.
Gender: females/males	9/6	9/6	NS.
Verbal intelligence ^a(^*M, SD*)	98.7 (8.9)	98.5 (10.1)	NS
Education, years (*M, SD*)	11.6 (1.2)	13.9 (1.6)	P < 0.001
Duration of any substance abuse, years (*M, SD*)	15.2 (6.1)		
Duration of opioid abuse, years (*M, SD*)	8.6 (4.1)		
Duration of withdrawal, days (*M, SD*)	9.6 (2.3)		
Short Opiate Withdrawal Scale score (*M, SD*)	9.8 (5.7) (n = 13)		

^a^Estimation based on WAIS-R Vocabulary score.

The dependence and other psychiatric diagnoses were done according to the Structured Clinical Interview (SCID) for DSM-IV axes I and II [38,39]. All patients met the DSM-IV criteria for opioid dependence. Nine patients also fulfilled the criteria for benzodiazepine dependence and four for cannabis dependence. One patient had all these three diagnoses. Self-reported recent month drug abuse was consistent with urine screening results. Table 2 shows recent month drug abuse in the patient group.

**Table 2:** Recent month drug abuse in patient group (number of patients)

	Main opioid abused	
	
	Buprenorphine 10	Heroin 5
Opioid only		1
Opioid with occasional benzodiazepine	3	
Opioid with frequent benzodiazepine	6	2
Opioid with frequent benzodiazepine and occasional cannabis		1
Opioid use with benzodiazepines		
Opioid with frequent cannabis without other substances of abuse	1	1

Note: Occasional = five to ten days of abuse. Frequent = more than ten days of abuse. All who had abused mainly heroin within recent month did it were intravenously. All but two abused buprenorphine intravenously.

During the inpatient period many participants with opioid dependence showed current mood or anxiety disorder symptoms. However, only two participants were classified as having other axis I diagnosis than substance-abuse. Both of these were depressive disorders not otherwise specified. When DSM-IV axis II diagnoses were evaluated, all patients met criteria for at least one personality disorder. The most common of these was the antisocial personality disorder, which was diagnosed in all except three, who also had some features of antisocial personality disorder.

### Cognitive tests

A battery of cognitive tests included tests of working memory, episodic memory, executive function, and fluid intelligence. All tests were administered according to standard instructions.

**Working memory **was measured by The Digit Span subtest from the Wechsler Memory Scale-Revised (WMS-R) and a computerized version of the Paced Auditory Serial Addition Task (PASAT) from the FORAMENRehab software package [40-42]. The Digit Span measures verbal working memory storage in relatively simple form. In the PASAT complex working memory functions are required: continuous storage of previous number, rapid arithmetical processing, and executive control of interference from previous items or from ongoing adding process. Presentation rate of a new number to be added with the previous one was one in every 1.6 second.

**Verbal memory **was measured by The delayed Logical Memory subtest of the WMS-R and the Rey Auditory Verbal Learning Test (the RAVLT) [40,43]. To avoid ceiling effect in the RAVLT only three learning trials of 15 word list were presented. The sum of the first three RAVLT trials was used as a parameter for immediate learning. **Visual memory **was measured by the Benton Visual Retention Test [44].

**Executive function **was measured using the modified Stroop task and the Ruff Figural Fluency Test [45,46]. The interference time of the Stroop task was calculated by subtracting reading time of 50 non-colored words from naming time of 50 colors printed in a different color than the one spelled by the letters by a procedure first described by Dodrill [47]. Thus, both color naming speed and interference from inhibition from not reading words are affecting the result. According to current research this procedure should be more prone to interference effect than subtracting from color naming [48]. The RFFT is a design fluency task measuring planning and fluency of action. A paper containing a series of squares with five dots in each one is presented. Then as many as possible unique figures are drawn by connecting at least two dots with straight line in each square.

**Fluid intelligence **was measured by the Culture Fair Intelligence Test (CFIT) also known as Cattell's Culture Fair test [49]. Version 2A was used. The CFIT includes a group of visuo-spatial reasoning tasks. The performance of the participant in these tasks reflects fluid or general intelligence needed in highly demanding novel problem solving situations. This test is sensitive to fluid intelligence deficit due to various origins. Dissociations between preserved standard intelligence measured by the Wechsler scales and by the poor CFIT scores have been shown in frontal lobe lesions as well as in normal ageing [30,50]

### Procedure

The psychiatric examination and diagnosis were done by trained psychiatrists at the detoxification unit. The day of cognitive testing was randomly chosen from days between 5 to 15 days from the last opioid dose. Tests were presented alternating between difficult and easy ones, and verbal and nonverbal ones, and memory and non-memory ones. To avoid fatigue, one pause was held during testing. Patients showing positive urine drug screening at initiation of withdrawal period were tested after negative drug screening for other drugs than those prescribed for them. On the test day morning at 8.00 patients completed the 10-item Short Opiate Withdrawal Scale (SOWS) [51] measuring withdrawal symptoms. All patients were using symptom relieving or other psychotropic medication on the day of testing. Medication variables of the test day are presented in Table 3. The brain MRI scans were evaluated by two neuroradiologists. A consensus of opinion was formed in each case. Analyses of MRI results are reported separately [52].

**Table 3:** Medication within last 24 h before testing in patient group

Medications used within 24 hours of test	Number of patients	Dose, range
Antidepressives	4 (26 %)	
Doxepine	2	50 – 100 mg
Sertraline	1	150 mg
Venlaflaxine	1	150 mg

Anxiolytics, sedatives and hypnotics (Benzodiazepines)	15 (100%)	
Diazepam	10	5 – 30 mg
Oxazepam	4	15 – 60 mg
Tematzepam *	4	20 – 40 mg
Zolpidem *	1	10 mg
Zopiclone *	1	15 mg

Neuroleptics †	10 (67%)	
Chlorprotixine/Truxal	1	75 mg
Promazine	9	100 – 200 mg
Melperone	3	75 mg

Other opioid withdrawal symptom relievers	15 (100%)	
Hydroxyzine	6	100 – 300 mg
Lofexidine	6	0.2 – 1.2 mg
Naproxen/Alpoxen	6	1000 – 1500 mg

^* ^Used as a hypnotic the night before testing. † Used with anxiolytic indications

### Statistical analysis

Analysis of variance (ANOVA) was used to compare the raw scores of each cognitive test. Statistical significance was set at 0.05 (two-tailed). P-values of 0.05–0.1 were considered as a statistical trend. Effect sizes were calculated with Cohen's d. Corrections for multiple comparisons were not applied due to seminal nature of this study and the limited power afforded by small number of participants. Group difference in education was not covaried for, because the assumption of similar linear relation between education and cognitive performance in both groups needed for analysis of covariance (ANCOVA) was not met. All participants with opioid dependence had started substance abuse in their early teen years. Once the substance abuse history begins, early-onset substance abusers start to skip school lessons at primary school, get poor grades, and only few of them get a diploma from secondary education. Thus, as a group, the participants with opioid dependence had less education than their VIQ comparable controls. Thus, group difference in education does not give as reliable estimate about their primary intellectual capacity than VIQ. Whenever participants with opioid dependence performed significantly worse than control participants correlations between cognitive performance and variables of interest, were analyzed by the Pearson product-moment correlation coefficient. Statistical analyses were done by SPSS statistical PC program, version 11.0.

## Results

Table 4 shows that participants with opioid dependence performed significantly worse than control participants in fluid intelligence, measured by the CFIT, in complex working memory, measured by the PASAT, and in one executive function test, the RFFT.

**Table 4:** Comparisons of individuals with opioid dependence and controls on cognitive measures using ANOVA

	**Controls (n = 15)**	**Individuals with Opioid Dependence (n = 15)**				
**Domain**	**Mean ± SD (CI)**^a^	**Mean ± SD (CI)**	***F***	**df**	**d**	***P***

**Test**						
Attention						
PASAT	47.5 ± 7.8 (43.2 – 51.8)	36.1 ± 10.1 (30.4 – 41.7)	12.00	1,28	1.26	0.002
WMS-R Digit Span	15.4 ± 3.8	14.9 ± 2.7	0.15	1,28	0.15	NS
Memory						
RAVLT, sum of learning trials 1–3	32.3 ± 6.1	28.9 ± 6.0	2.64	1, 28	0.56	NS
RAVLT, delayed recall	10.7 ± 2.8	9.0 ± 2.8	2.65	1, 28	0.61	NS
WMS-R Logical Memory, immediate	28.0 ± 5.5	25.9 ± 8.1	0.72	1, 28	0.30	NS
WMS-R Logical Memory, delayed recall	25.1 ± 6.6	22.3 ± 7.3	1.20	1, 28	0.40	NS
BVRT, number of right figures	7.4 ± 1.3	6.8 ± 1.6	0.88	1, 28	0.41	NS
Executive function						
Stroop, modified interference time	24.5 ± 12.0	25.1 ± 8.8	0.30	1, 28	0.12	NS
RFFT, unique designs	86.3 ± 22.6 (73.9 – 98.8)	68.1 ± 21.2 (43.2 – 51.8)	5.22	1, 28	0.83	0.03
RFFT, perseverative errors	2.8 (2.6)	3.4 (2.9)	0.34	1,28	0.22	NS.
Fluid intelligence						
CFIT	34.0 ± 3.8 (32.5 – 36.7)	30.4 ± 4.2 (28.0 – 32.8)	7.97	1, 27	0.90	0.009

^a ^CI = confidence interval

Note: CFIT = Culture Fair Intelligence Test; PASAT = Paced Auditory Serial Addition Task; WMS-R = Wechsler Memory Scale-Revised; RAVLT = Rey Auditory Verbal Learning; BVRT = Benton Visual Retention Test; RFFT = Ruff Figural Fluency Test.

**Figure 1:**
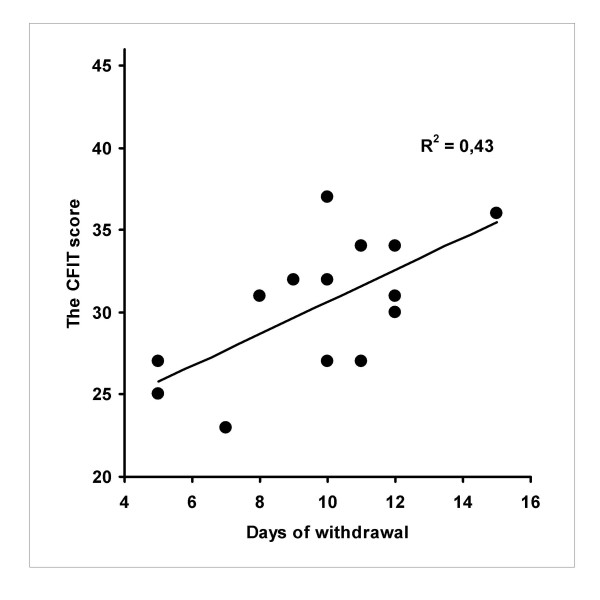
**Correlation between fluid intelligence test performance and days of withdrawal among individuals with early abstinence from opioid dependence (N = 14) **^a ^Note: CFIT = Culture Fair Intelligence Test. ^a ^= One outlier performance (17 points) highly discordant to his other performance was dismissed due to poor collaboration during the CFIT administration.

**Figure 2:**
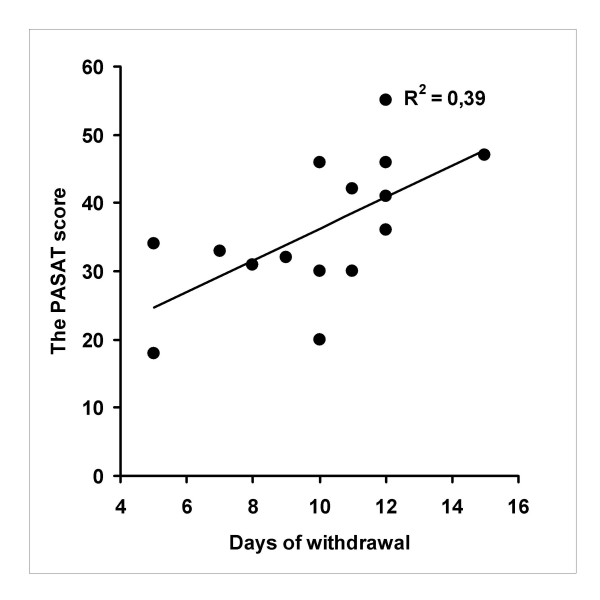
**Correlation between complex working memory test performance and days of withdrawal among individuals with early abstinence from opioid dependence **Note: PASAT = Paced Auditory Serial Addition.

Analysis of correlations between inferior cognitive performance and days of withdrawal showed statistically significant results with fluid intelligence measured by the CFIT performance and with complex working memory measured by the PASAT (R = .65, P = 0.009 and R = .63, P = 0.01, respectively). Figures 1 and 2 depict these correlations. However, reduced figural fluency performance in the RFFT and days of withdrawal showed no association (R = -.01, NS). The highest correlations between years of opiate abuse or years of any substance abuse and between inferior cognitive performances were found for the RFFT performance -.23 and -.31; respectively: Both of these were statistically non-significant. The correlations between the SOWS score and inferior cognitive performance ranged from 0.08 to -0.13 (NS).

## Discussion

Early abstinent opioid dependent individuals performed worse than normal controls in complex working memory, executive function, and fluid intelligence. Thus, the results support our main hypothesis of reduced cognitive performance in these areas during early opioid abstinence. The effect sizes of the group differences in these areas are similar or higher than the ones found in studies concerning current drug abuse [53]. The high positive correlations that were found between fluid intelligence performance or complex working memory performance and days of withdrawal support the hypothesis of transient cognitive deficit. Although correlations do not imply causality, they may give impetus for more detailed considerations.

If neurocognitive deficits related to opioid abuse are considered to reflect a rather permanent neurotoxicity of opioid abuse, then the effect of abuse period under which the study was done (ongoing abuse vs. early abstinence vs. late abstinence) should be small. However, as reviewed in the background section neural dysregulations are pronounced during early opioid abstinence; and this is likely to affect cognition. This conclusion is supported by comparing our complex working memory results to the results by Mintzer et al study in which a two-back working memory task was employed [14]. In their study opioid dependent patients treated with opioid agonist methadone showed working memory deficit, whereas patients with nine months of opioid abstinence showed normal performance. Thus, there is some evidence that individuals with current opioid use or under early opioid abstinence show complex working memory deficit, whereas individuals who have reached late opioid abstinence do not show similar deficit. This is in agreement with the suggestion that neurocognitive deficits related to several drugs of abuse including opioids should be seen as recoverable limitations of neuronal plasticity rather than as more permanent "lesion effect" [54].

The relevance of neurocognitive study results comes often from dissociations found between different functions. In this study three dissociations merit further consideration. First, early opioid abstinent patients were inferior to controls in fluid intelligence task though the groups were matched for VIQ. According to functional neuroimaging studies this indicates deficiencies in frontoparietal networks needed in several demanding cognitive task [55]

The second dissociation was found between deficient performance in complex working memory task, the PASAT, and normal or nearly normal episodic memory performance. Against our expectations there were no group differences in immediate or delayed free recall tasks. Thus, it is possible that neural dysregulations in hippocampus during opioid withdrawal may not affect episodic memory.

It has been suggested that stress system induced episodic memory impairment needs more chronic stress system abnormality than working memory impairment; and this would hold especially among young adults [56,57]. During early opioid abstinence high stress system activation as shown by elevated cortisol level is common. It is especially pronounced among individuals with antisocial personality disorder [35]. Therefore, as nearly all patients studied were young adults (mean 31.5 years) and most of them (12/15) had antisocial personality disorder, elevated stress system activation during early opioid abstinence may be related to the dissociation observed between complex working memory and episodic memory.

The third dissociation was found between deficient complex and normal simple working memory performance: the PASAT and the Digit Span, respectively. In the PASAT both storage and central executive components of working memory are needed [58]. The Digit Span task demands especially storage of the several items and central executive is involved to a lesser degree. Thus, we suggest that central executive component of working memory is impaired during early opioid abstinence while storage is intact.

There are some studies indicating that executive function deficit may be found during late opioid abstinence as well [12,13]. This idea is in agreement with zero-correlation found between days of withdrawal and reduced figural fluency performance. In addition, the non-significant negative correlations between figural fluency performance and duration of opioid abuse or any substance abuse (-.23 and -.31) are in line with earlier research showing negative association between opioid abuse severity and figural fluency performance [59]. A larger sample study concerning the relationship between opioid abuse or other substance abuse variables and fluency performance is warranted.

The positive correlations that were between impaired complex working memory or fluid intelligence performances and days of withdrawal are in agreement with the idea that the observed deficits in these domains may be partly transient. It is known that high cortisol levels may associate with working memory deficit [23,24]. It is also known that during early opioid abstinence cortisol levels are elevated but then start to normalize during second week of abstinence [33]. Thus, we suggest that cognitive deficit during early opioid abstinence may partly relate to high brain stress system activation during that time, and therefore is partly transient. In agreement with this idea, a recent experimental study showed that chronic stress system elevation leads to transient – not permanent – dysfunction – of prefrontal dopamine system [60]. The authors suggest that this may negatively affect working memory. Interestingly, working memory and fluid intelligence are highly associated [61].

Finally, the correlations found between opioid withdrawal symptoms as measured by the SOWS and cognitive performances were practically zero. It is possible that the SOWS reflects mostly peripheral aspects of opioid withdrawal syndrome.

### Clinical implications

Higher order cognition and prefrontal cortex function are closely related. It has been suggested that when prefrontal cortex function gets "off line" in prolonged stressful situations more habitual responses start to regulate behavior [62]. Thus, reduced higher order cognition and impulsive behavior during early opioid abstinence are likely to be associated. Therefore, screening of higher order cognitive functions by highly sensitive task like the PASAT could be used for treatment planning. In addition, pharmacological and behavioral interventions to improve working memory performance may improve opioid withdrawal outcomes as well [63,64].

### Limitations

The small number of participants reduces the power to detect mild to moderate cognitive deficits. Previous benzodiazepine abuse or cannabis abuse that was common among our patient group may also affect the results of this study. Long-term cannabis abuse and benzodiazepine abuse both have adverse effect on cognitive function [65,66]. Current benzodiazepine medication at test was common. In normal population benzodiazepines have adverse affect on several cognitive functions. On the other hand, noradrenaline agonist lofexidine, which was given to the patients of this study, may improve reduced working memory performance [63]. In our sample the frequency of personality disorders, and especially antisocial personality disorder was more common than known frequencies of these disorders among substance abusers. This also should be taken into account when comparing our results with other studies. The group matching procedure of our study was based on VIQ whereas in most other studies the matching is based on education. Though matching for VIQ or premorbid IQ is not totally exceptional in opioid abuse studies [6,11], this should be taken into account when comparing our results with other studies. Finally, the suggestion for transient cognitive deficit due to high stress system activation is very preliminary. Longitudinal studies with relevant cognitive and neuroendocrine variables may determine if suggested associations between these variables exist.

## Conclusion

Fluid intelligence, working memory, and executive function deficit observed in opioid withdrawal patients during first or second week from detoxification implicates a rather general cognitive deficit. We suggest that cognitive deficit during early opioid abstinence may be associated with known opioid withdrawal related neural dysregulations in the prefrontal cortex. Fortunately, relatively rapid recovery of cognitive function during opioid abstinence seems possible. Yet, the role of cognitive control during the narrow gateway to long-term opioid abstinence needs further studies.

## Competing interests

The author(s) declare that they have no competing interests.

## Authors' contributions

PR was the main investigator collecting neuropsychological data. He was principally responsible for preparing the manuscript and performed statistical analysis. Together with authors TA, RK, and SK, he conceived the idea of this study.

HK and SK participated in the design of the study and also in writing the manuscript.

TA and RK carried out MRI investigations.

OJ and VP together with collaborators carried out psychiatric investigations.

All authors read and accepted the manuscript.

## Pre-publication history

The pre-publication history for this paper can be accessed here:


